# HK2 contributes to the proliferation, migration, and invasion of diffuse large B-cell lymphoma cells by enhancing the ERK1/2 signaling pathway

**DOI:** 10.1515/biol-2022-0726

**Published:** 2023-10-14

**Authors:** Hongcan Zhao, Guoqian Xiang, Tingjun Shao, Minmin Wang, Weijian Dai

**Affiliations:** Department of Laboratory Medicine, Affiliated Hangzhou First People’s Hospital, Zhejiang University School of Medicine, No. 261 Huansha Road, Shangcheng District, Zhejiang, China

**Keywords:** diffuse large B lymphoma, Hexokinase, ERK1/2, FR180204

## Abstract

Hexokinase 2 (HK2) has been associated with carcinogenic growth in numerous kinds of malignancies as essential regulators during the processing of glucose. This study aimed to explore the effects of HK2 on diffuse large B-cell lymphoma (DLBCL) cells via the ERK1/2 signaling. Expressions of HK2 and ERK1/2 were examined in DLBCL cell lines using quantitative reverse transcription polymerase chain reaction and western blotting. HK2 and ERK1/2 were attenuated through HK2 small-interfering RNA (siRNA) and ERK inhibitor FR180204, respectively, in U2932 and SU-DHL-4 cells. Cell Counting Kit-8, clone formation, transwell, and flow cytometry assays were used in evaluating the effects of HK2 and ERK1/2 on cell proliferation, migration, and apoptosis. Moreover, a xenograft model was created to assess the roles of HK2 *in vivo*. HK2 and ERK1/2 were evidently up-regulated in DLBCL cell lines. HK2 knockdown and FR180204 markedly suppressed the proliferation and clonogenesis of U2932 and SU-DHL-4 cells and promoted cell apoptosis *in vitro*. We also found that HK2 silencing suppressed tumor growth *in vivo*. Notably, HK2 knockdown inactivated the ERK1/2 signaling pathway both *in vitro* and *in vivo*. These data indicate that inhibition of HK2 may suppress the proliferation, migration, and invasion of DLBCL cells, partly via inhibiting the ERK1/2 signaling pathway.

## 1 Introduction

Lymphoma is a malignancy originating from the lymphatic hematopoietic system, with over 67 subtypes, including Hodgkin’s lymphoma (HL) and non-Hodgkin’s lymphoma (NHL). Among these, HL includes 6 subtypes and NHL includes 61 subtypes; thus, most lymphomas are NHLs [1]. It is estimated that 544,000 new cases of NHL were detected worldwide in 2020, with roughly 260,000 people dying as a result of the disease [2]. Diffuse large B-cell lymphoma (DLBCL) is the most common subtype of NHL, accounting for approximately 30–40% of all NHL cases [3]. Rituximab, cyclophosphamide, doxorubicin, vincristine, and prednisone (R-CHOP) are the first-line treatment options for DLBCL. Most patients experience remission after treatment, although approximately 10–15% of patients develop primary resistance and 20–25% of patients experience relapse following initial treatment [4,5]. Hence, establishing a prognostic evaluation system and finding new therapeutic targets for DLBCL represent rigorous clinical challenges for researchers.

DLBCL is a highly heterogeneous NHL, as reflected in its morphology, immunohistochemical phenotype, molecular genetics, involved sites, chemotherapy response, and survival rate [6,7,8]. Different subtypes are crucial for treatment selection and prognosis. Therefore, in recent years, the classification and prognosis of DLBCL have been extensively explored, and fruitful results have been obtained. As such, various types of assessment systems and markers closely related to DLBCL prognosis have been confirmed. These findings provided the basis for judging patient prognosis and selecting effective interventions for high-risk patients as well as for establishing individualized, precise, and standardized treatments. In addition, more comprehensive prognostic models can allow for the early identification of high-risk patients, enabling clinicians to select the appropriate treatment plan in advance and improve the remission rate and survival duration of patients. In this context, further exploration of the molecular changes and drug resistance mechanisms of DLBCL can contribute to the development of targeted drugs for this malignancy in the future, achieving significant therapeutic efficacy.

Hexokinases (HKs; or glucokinases) are involved in the first step of glucose metabolism, wherein glucose or glycogen is phosphorylated to glucose 6-phosphate. To date, four HK isoenzymes, namely HK1, HK2, HK3, and HK4, have been identified [9]. Of these, HK2 is primarily detected in embryos and is highly expressed in very few adult tissues, such as fat and skeletal muscles [10]. In recent years, many studies have shown that HK2 is highly expressed in breast cancer, liver cancer, glioma, and esophageal cancer and can promote tumor progression through energy metabolism and anti-apoptotic signaling pathways [9,11,12]. For instance, Bhalla et al. [13] found that HK2 is a key molecule that mediates hypoxia to promote DLBCL proliferation. Gao et al. [14] found that TNFα-YAP/p65 can synergistically regulate HK2 expression and promote breast cancer cell migration. Li et al. [15] and Zhang et al. [16] found that HK2 is closely related to the resistance of ovarian cancer to paclitaxel and cisplatin. Yuen et al. [17] reported that hypoxia can regulate the expression of Warburg effect-related molecules, including HK2, in cancer stem cells, thereby promoting malignant biological behaviors, such as tumor drug resistance [17]. In addition, Lu et al. [18] showed that the covalent lysine (K)-specific demethylase 1A (KDM1A) inhibitor ORY-1001 regulates the Warburg effect by controlling HK2 expression, which in turn inhibits lung cancer cell proliferation and clone formation but promotes apoptosis [18]. Agnihotri et al. [19] found that HK2 showed abnormally high expression in glioma and may serve as a potential therapeutic target [19]. By constructing a direct interacting protein network, HK2 was found to directly interact with EGFR, BUB3, and CircCDKN2B-AS1, which are proteins involved in the malignant progression of various tumors [20,21,22]. Therefore, HK2 likely promotes the malignant progression of DLBCL through signaling pathways other than energy metabolism. Specifically, ERK is an early member of the MAPK family, and its isomers ERK1 and ERK2 are involved in the regulation of cell proliferation, differentiation, apoptosis as well as other physiological processes [23]. From these reports, HK2 may be closely associated with malignant progression and serve as a potent therapeutic target for DLBCL.

Apoptotic suppression is important for rapid tumor growth, and apoptosis involves the expression and regulation of several genes, signaling pathways, and proteins. The Bcl-2 protein family constitutes many pro- and anti-apoptotic proteins [24]. However, the link between HK2 and lymphoma, specifically DLBCL, remains unclear, and its roles in the proliferation and metastasis of this malignancy from a prognostic viewpoint have rarely been reported. In the present study, we confirmed that HK2 is upregulated in DLBCL cells. HK2 knockdown inhibited the proliferation, migration, and invasion of DLBCL cells *in vitro* and restricted tumor growth *in vivo*, and these effects were associated with the suppression of the ERK1/2 signaling pathway. These findings offer novel insights into the oncogenic roles of HK2 in DLBCL, which may further our understanding of the pathogenesis of and therapeutical strategies for this malignancy.

## 2 Materials and methods

### 2.1 Cell culture and transfection

Human normal B-cell line HMy2.CIR was preserved in our laboratory and DLBCL TMD8, OCI-Ly3, U2932, OCI-Ly1, OCI-Ly7, SU-DHL-4, and SU-DHL-6 cells were purchased from the American Type Culture Collection (Manassas, VA, USA). HMy2.CIR, OCI-Ly3, and OCI-Ly7 cells were cultured in Iscove’s modified Dulbecco’s medium (IMDM) (Gibco, USA) containing 10% fetal bovine serum (FBS), 100 μg mL^−1^ streptomycin, and 100 U mL^−1^ penicillin (HyClone). The rest of the cells were maintained in RPMI 1640 medium with 10% FBS. All cells were cultivated in a humidified incubator at 37℃ with 5% CO_2_. To knockdown HK2 in the cells, siRNA sequences against HK2 (si-HK2 #1: 5′-GGAGATGGAGAAAGGGCTT-3′; si-HK2 #2: 5′-GCGCATCAAGGAGAACAAA-3′) or negative control (si-NC: 5′-AGACTTCCTCACCTCCTTCT-3′) were synthesized by Genepharm Co. Ltd. (Shanghai, China). All transfections were performed using Lipofectamine 3000 (Invitrogen, USA) according to the manufacturer’s procedures. The transfection efficiency was assessed with quantitative reverse transcription polymerase chain reaction (qRT-PCR).

### 2.2 Cell proliferation assay

Forty-eight hours after transfection, 3 × 10^3^ HK-2-silenced U2932 and SU-DHL-4 cells were seeded into 96‐well plates. Moreover, the ERK inhibitor F180204 (5 μm) was supplemented to the medium, and then, the Cell Counting Kit-8 (CCK-8) solution (Sigma-Aldrich, USA) was added to each well after 24, 48, 72, and 96 h of incubation. Next, absorbance at 450 nm was recorded using a microplate reader (Molecular Devices, USA). For cell colony assay, 6 × 10^2^ HK-2-silenced or F180204-treated U2932 and SU-DHL-4 cells were cultured on 6-well plates for 14 days. Thereafter, the cells were fixed and stained with 0.1% crystal violet for 30 min. Finally, cell colonies comprising over 50 cells were photographed and counted under a light microscope (AE2000, Motic).

### 2.3 Cell apoptosis assay

Annexin V-FITC apoptosis detection kit was purchased from Beyotime (Shanghai, China) to assess the apoptosis of HK-2-silenced or F180204-treated U2932 and SU-DHL-4 cells following the manufacturer’s instructions. After being harvested and centrifuged, the cells were incubated with 500 μL of Annexin V-FITC-binding solution and then stained with 5 μL of Annexin V-FITC and 10 μL of PI. Thereafter, apoptosis was evaluated using a flow cytometer and analyzed with FlowJo (V10.8.1, BD Biosciences, USA).

### 2.4 Transwell assays

For cell migration assay, 1 × 10^4^ HK-2-silenced or F180204-treated U2932 and SU-DHL-4 cells were cultured with 200 μL of a serum-free medium in the upper chamber of a transwell system (Corning, NY, USA). For cell invasion assay, the transwell chambers were pretreated with Matrigel™, and an equal number of cells were inoculated in the upper chamber. Then, 500 μL of RPMI 1640 medium containing 20% FBS was added to the lower chambers. After removing non-adherent cells in the upper chamber with cotton-tipped swabs, the cells that invaded and migrated to the lower chambers were fixed with 4% paraformaldehyde and stained with 0.1% crystal violet. Finally, the invading and migrating cells were captured and counted under a light microscope.

### 2.5 qRT-PCR assays

Total RNA from HMy2.CIR and DLBCL cells was extracted with TRIzol® reagent (Invitrogen) and reverse-transcribed into cDNA using the Transcriptor First-Strand cDNA Synthesis Kit (Takara Biotechnology, Dalian, China). Subsequently, PCR amplification was performed using SYBR Premix Ex Taq II Kit (Takara) on a CFX Connect Real-Time PCR Detection System (Bio-Rad, USA) to measure the expression levels of HK2. Glyceraldehyde 3-phosphate dehydrogenase (GAPDH) was used as an internal control. The primers used in the present study were synthesized by Sangon Biotech (Shanghai, China): HK2 forward 5′-GAGCCACCACTCACCCTACT-3′; HK2 reverse 5′-CCAGGCATTCGGCAATGTG-3′; GAPDH forward 5′-TGTGGGCATCAATGGATTTGG-3′; and GAPDH reverse 5′-ACACCATGTATTCCGGGTCAAT-3′. The relative expression levels of HK2 were calculated using the 2^−ΔΔCt^ method [25].

### 2.6 Western blotting

Total protein was extracted from HMy2.CIR and DLBCL cells using ice-cold RIPA lysis buffer (Solarbio, Beijing, China) supplemented with proteinase inhibitors (Beyotime, China). Next, protein concentration was assessed using the BCA protein assay kit (Beyotime, China). Equal amounts (25 μg) of protein extracts were separated with 10% sodium dodecyl sulfate–polyacrylamide gel electrophoresis and transferred onto polyvinylidene fluoride (PVDF) membranes (Millipore, Burlington, MA, USA). The PVDF membranes were blocked with 5% nonfat milk for 2 h at room temperature, following incubation with the primary antibodies against HK2 (1:1,000, BF0283, Affinity), ERK1/2 (1:2,500, AF0155, Affinity), phospho-ERK1/2 (Thr202/Tyr204) (1:1,000, AF1015, Affinity), Bax (1:2,000, ab182733, Abcam), Bcl-2 (1:1,000, AF6139, Affinity), caspase-3 (1:500, ab13847, Abcam), E-cadherin (1:2,000, 20874-1-AP, ProteinTech), N-cadherin (1:1,000, AF6139, Affinity), and GAPDH (1:5,000, AF7021, Affinity) at 4°C overnight. After washing three times with TBST, the membranes were incubated with an anti-rabbit or anti-mouse HRP-conjugated secondary antibody for 1 h. Finally, bands were detected using an enhanced chemiluminescence (Beyotime, China) system and analyzed using ImageJ (v1.8.0, National Institutes of Health, USA).

### 2.7 Tumor xenograft models

To identify the roles of HK2 in DLBCL tumor growth *in vivo*, a xenograft mouse model was established in the present study. In brief, 5 × 10^6^ HK2-knockdown U2932 cells and the corresponding control cells were subcutaneously injected into the flanks of 4-week-old BALB/c nude mice (Shanghai SLAC Laboratory Animals Ltd., China). Tumor volume was recorded every 5 days using a vernier caliper and calculated using the formula: volume (mm^3^) = width^2^ × length/2. After 30 days, the xenografts were collected and weighed. Additionally, the xenograft tissues were subjected to hematoxylin and eosin (HE) staining, terminal-deoxynucleoitidyl transferase-mediated nick end labeling (TUNEL) assay, and immunohistochemical (IHC) analysis.


**Ethical approval:** The research related to animal use has been complied with all the relevant national regulations and institutional policies for the care and use of animals and was approved by the Ethics Committee of Zhejiang Eyong Pharmaceutical Research and Development Center (certificate no. SYXK (Zhe) 2021-0033, Hangzhou, China).

### 2.8 HE staining, TUNEL assay, and IHC analysis

For HE staining, the xenograft tissues were fixed, dehydrated, paraffin-embedded, sectioned, and stained with HE (Solarbio, China). HE-stained images were captured using a microscope at 200× magnification. For the TUNEL assay, 4-μm-thick paraffin-embedded xenograft tissue sections were dewaxed, rehydrated, incubated with proteinase K, incubated in 3% H_2_O_2_, permeabilized with 0.5% Triton X-100, and incubated with a TUNEL reaction mixture for 1 h at 37°C in the dark. Next, the sections were incubated with 3,3′-diaminobenzidine (DAB; Beyotime, China) for 5 min, and then, nuclei were counterstained with hematoxylin (Solarbio, China). TUNEL staining was investigated, and photographs were scanned using a microscope at 400× magnification. For IHC analysis, the xenograft tissues were fixed, paraffin-embedded, sectioned, dewaxed, exposed to antigens, and quenched for endogenous peroxidase activity. Then, the sections were incubated with 10% goat serum for 30 min at room temperature, followed by anti-Ki67 (1:100, AF0198, Affinity), anti-caspase 3 (1:100, 19677-1-AP, ProteinTech), anti-N-cadherin (1:7,500, 66219-1-Ig, ProteinTech), anti-HK2 (1:100, DF6176, Affinity), anti-E-cadherin (1:200, ab231303, Abcam), and anti-p-ERK (1:200, 4370T, Cell Signaling Technology) overnight at 4°C. Thereafter, the sections were washed with PBS and incubated with an appropriate HRP-labeled secondary antibody for 30 min at 37°C. The slides were visualized using DAB staining and hematoxylin counterstaining. Finally, images were obtained with a microscope at 200× magnification and semi-quantitatively analyzed using ImageJ.

### 2.9 Statistical analysis

All results are presented as mean ± standard deviation. Statistical analysis was performed using Student’s unpaired *t*-test. A *P* < 0.05 was considered statistically significant.

## 3 Results

### 3.1 HK2 upregulation in DLBCL cell lines and its association with DLBC prognosis

**Figure 1:**
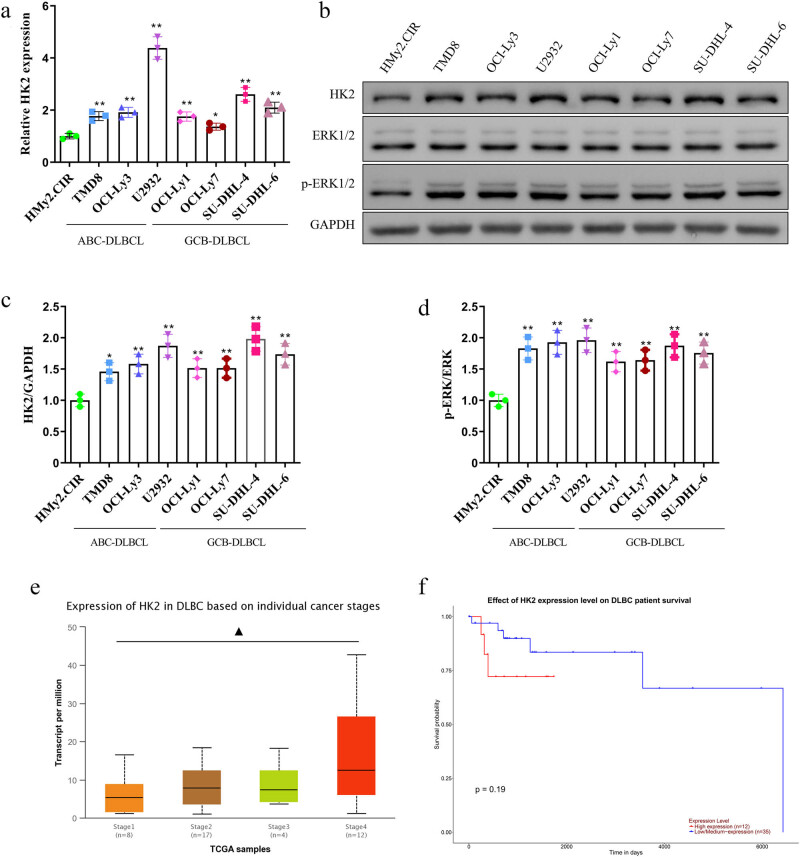
HK2 was overexpressed in DLBCL cell lines. (a) HK2 expression in normal (Hmy2.CIR) and DLBCL (TMD8, OCI-Ly3, U2932, OCI-Ly1, OCI-Ly7, SU-DHL-4, and SU-DHL-6) cells were measured using qRT-PCR. (b–d) Protein expression levels of HK2, p-ERK1/2, and ERK1/2 were assessed using western blotting. The University of Alabama at Birmingham Cancer (UALCAN) data analysis portal was used to analyze (e) the expression levels of HK2 at individual cancer stages and (f) overall survival of patients with DLBCL. *P < 0.05 and **P < 0.01 versus the Hmy2.CIR group. ^▲^ *P* < 0.05 Stage 1-versus-Stage 4 (*P*-value: 2.502100 × 10^−2^). ABC-DLBCL: activated B-cell-like (ABC) subtype of diffuse large B-cell lymphoma (DLBCL); GCB-DLBCL: Germinal center B-cell-like (GCB) subtype of diffuse large B-cell lymphoma (DLBCL).

To elucidate the roles of HK2 in the progression of DLBCL, the human normal B-cell line HMy2.CIR and the DLBCL cells TMD8, OCI-Ly3, U2932, OCI-Ly1, OCI-Ly7, SU-DHL-4, and SU-DHL-6 were used, and the expression levels of HK2 in these cells were explored with qPCR and western blotting. Both transcript and protein expression levels of HK2 were significantly higher in DLBCL cells than those in HMy2.CIR cells, particularly U2932 and SU-DHL-4 cells (Figure 1a–d). Therefore, HK2 silencing was investigated in U2932 and SU-DHL-4 cell lines. Interestingly, HK2 levels increased gradually with the progression of cancer stages in patients with DLBCL (Figure 1e). Furthermore, higher HK2 expression in patients with DLBCL was associated with worse overall survival, although no significant difference was noted in the UALCAN database [26] (Figure 1f). Therefore, HK2 may act as a potential therapeutic target for DLBCL.

### 3.2 HK2 knockdown inhibits U2932 and SU-DHL-4 cell proliferation

**Figure 2:**
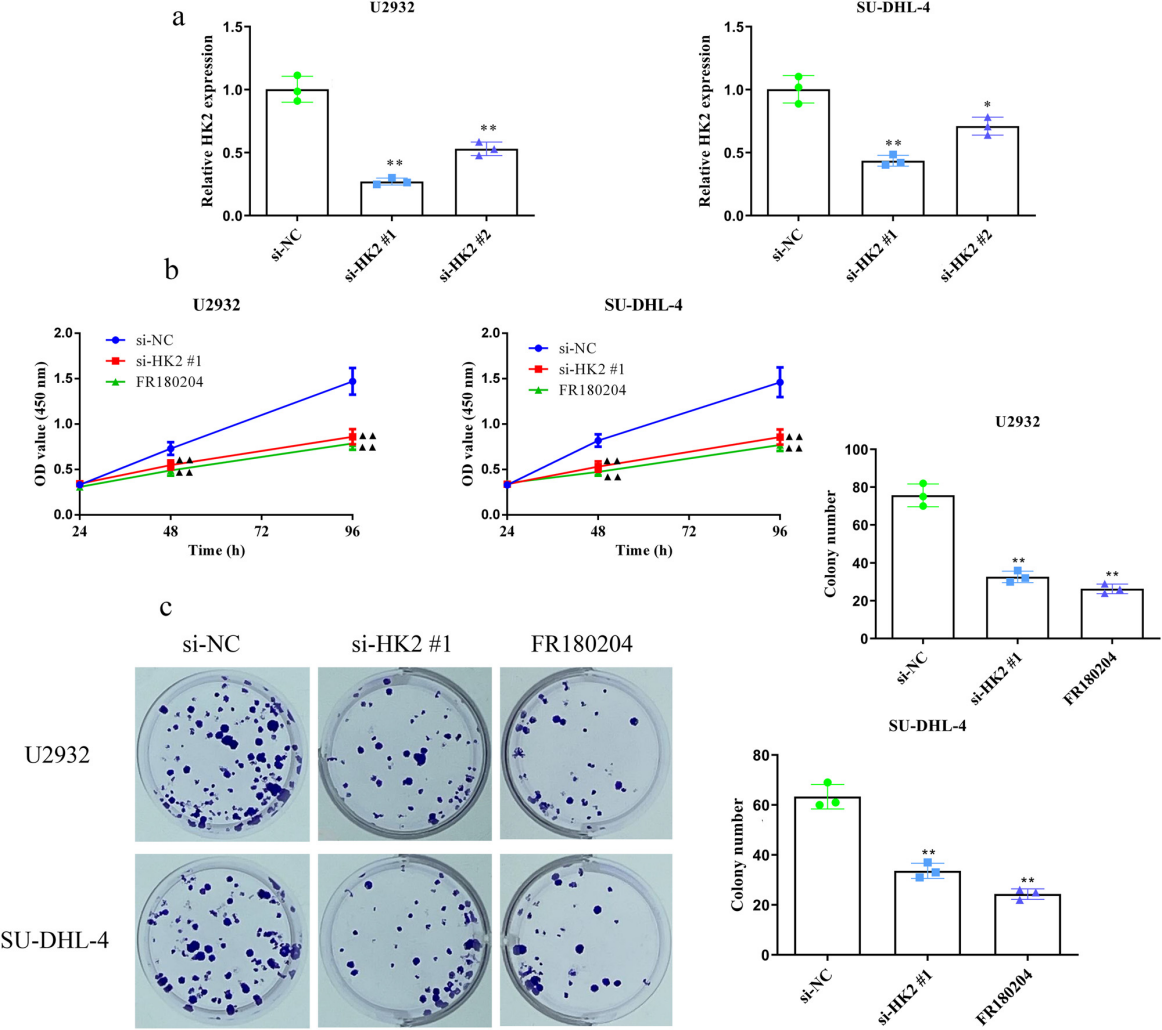
HK2 knockdown inhibits U2932 and SU-DHL-4 cell proliferation *in vitro*. (a) HK2 was knocked down in U2932 and SU-DHL-4 cells using siRNAs. (b) The viability of HK2-silenced and FR180204-treated (5 μM) U2932 and SU-DHL-4 cells was assessed using the CCK-8 assays. (c) Colony formation assays were used to assess the clone numbers of HK2-silenced and FR180204-treated U2932 and SU-DHL-4 cells. *P < 0.05 and **P < 0.01 versus the si-NC group.

To further identify its effects, we knocked down HK2 in U2932 and SU-DHL-4 cells. As shown in Figure 2a, RT-PCR analysis revealed that si-HK2 #1 and si-HK2 #2 significantly silenced HK2 levels compared with si-NC in U2932 and SU-DHL-4 cells, and the silencing effect was particularly strong for si-HK2 #1. Therefore, we selected si-HK2 #1 for subsequent experiments. HK2 silencing significantly suppressed U2932 and SU-DHL-4 cell proliferation and colony formation (Figure 2b and c). Similar inhibitory effects of the ERK inhibitor FR180204 were noted on DLBCL cell growth.

### 3.3 HK2 knockdown inhibits U2932 and SU-DHL-4 cell mobility

**Figure 3:**
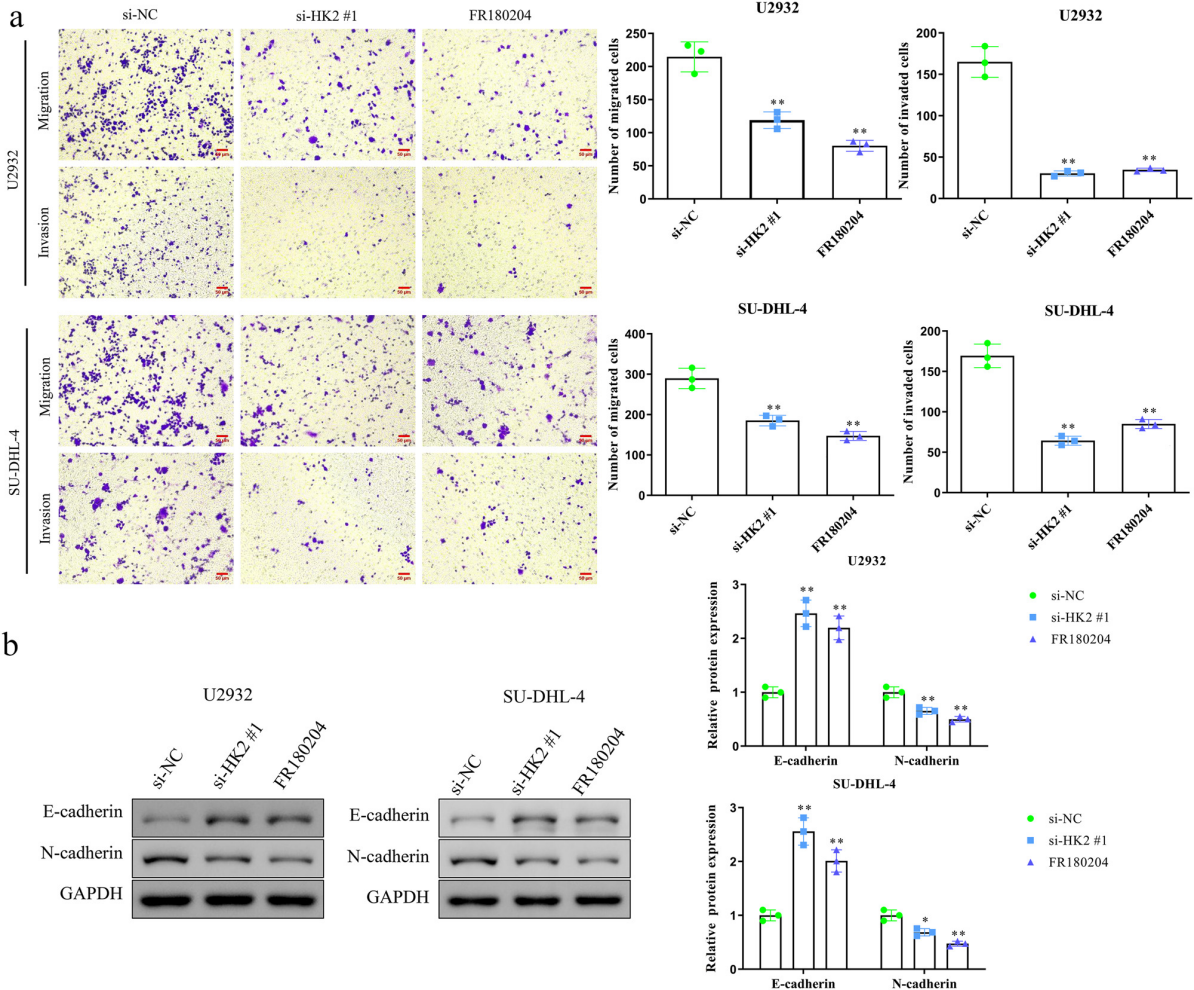
HK2 knockdown inhibits U2932 and SU-DHL-4 cell migration and invasion *in vitro*. (a) Transwell assays were used to analyze the effects of HK2 knockdown on the migration and invasion of U2932 and SU-DHL-4 cells. (b) Western blotting was used to assess the expression levels of E-cadherin and N-cadherin in HK2-silenced and FR180204-treated U2932 and SU-DHL-4 cells. *P < 0.05 and **P < 0.01 versus the si-NC group.

Transwell migration and invasion assays revealed that the number of migrating and invading cells was decreased in HK2-knockdown and FR180204-treated groups compared with those in the si-NC group (Figure 3a). Epithelial–mesenchymal transformation (EMT) contributes to cancer cell migratory and invasive abilities; therefore, we measured the expression levels of EMT-related proteins. Western blotting demonstrated that HK2 knockdown and FR180204 treatment increased E-cadherin levels but decreased N-cadherin levels in U2932 and SU-DHL-4 cells (Figure 3b).

### 3.4 HK2 knockdown promotes cell apoptosis of U2932 and SU-DHL-4

**Figure 4:**
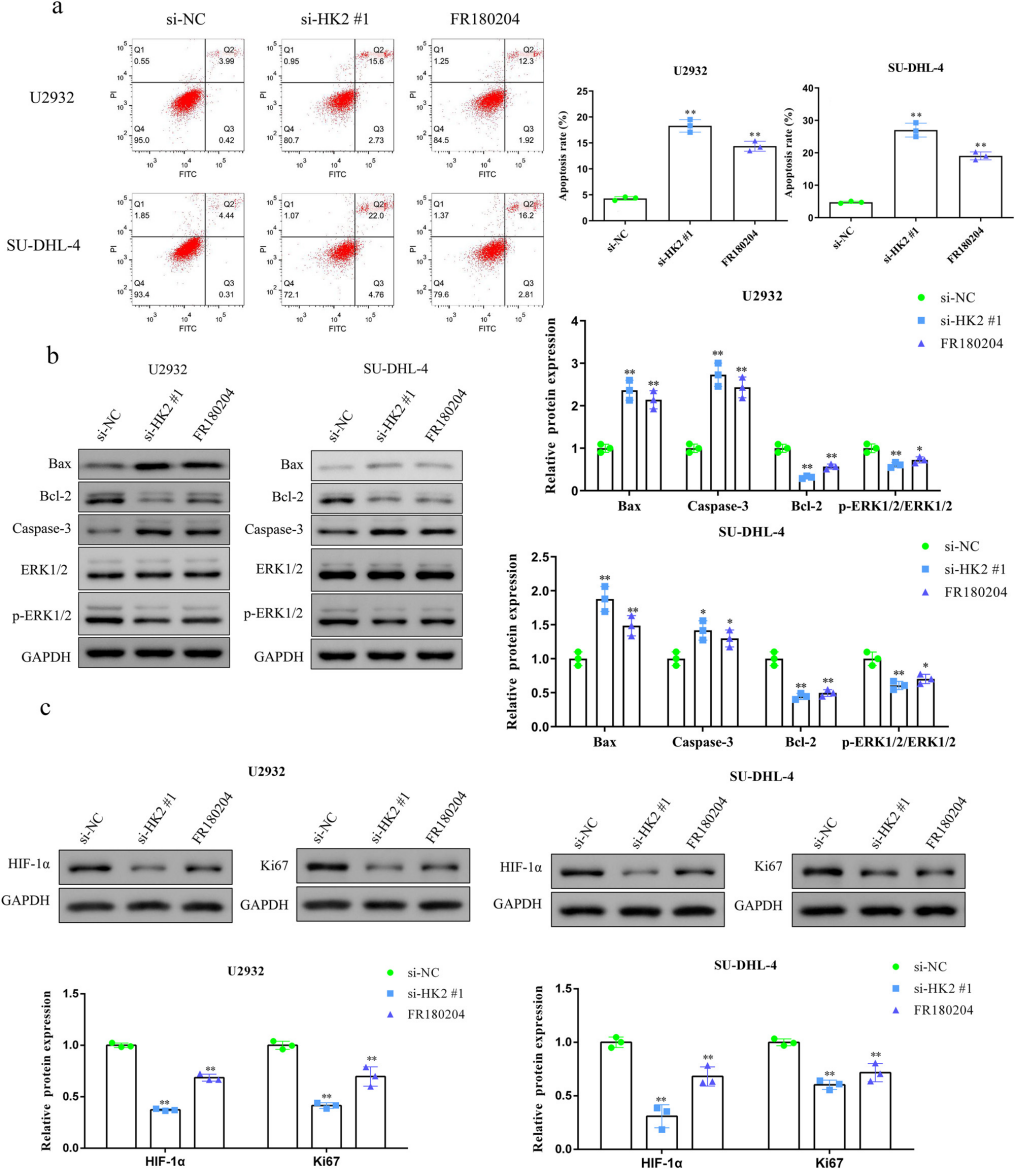
HK2 knockdown promotes U2932 and SU-DHL-4 cell apoptosis *in vitro*. (a) Flow cytometry assay was employed to measure U2932 and SU-DHL-4 cell apoptosis. (b) Representative blots and statistical analysis of Bax, Bcl-2, caspase-3, ERK1/2, and p-ERK1/2 in HK2-silenced and FR180204-treated U2932 and SU-DHL-4 cells. (c) Representative blots and statistical analysis of HIF-1α and Ki67 in HK2-silenced and FR180204-treated U2932 and SU-DHL-4 cells. *P < 0.05 and **P < 0.01 versus the si-NC group.

As shown in Figure 4a, HK2 knockdown and FR180204 treatment significantly promoted U2932 and SU-DHL-4 cell apoptosis. Next, we investigated whether HK2 affected U2932 and SU-DHL-4 cell apoptosis by regulating the expression of apoptosis-related proteins. HK2 knockdown increased protein levels of Bax and caspase-3 but decreased protein levels of Bcl-2 in U2932 and SU-DHL-4 cells. To examine the vital role of the ERK signaling pathway in DLBCL pathogenesis, we further assessed whether HK2 regulated this pathway. Western blotting revealed that p-ERK levels were significantly decreased in U2932 and SU-DHL-4 cells with HK2 knockdown (Figure 4b). Moreover, analogous effects were observed following the FR180204 treatment of U2932 and SU-DHL-4 cells. Additionally, hypoxia-inducible transcription factor 1α (HIF-1α) could bind with the promoter of HK2 and promote its transcription to increase glycolytic flux for promoting tumor survival and growth [27,28]. Western blot analysis showed that HK2 knockdown and FR180204 treatment rescued the HIF-1α and Ki67 proteins in U2932 and SU-DHL-4 cells (Figure 4c). These findings demonstrated that HK2 may serve as a positive regulator of the ERK pathway in DLBCL.

### 3.5 HK2 knockdown inhibits DLBCL tumor growth *in vivo*

**Figure 5:**
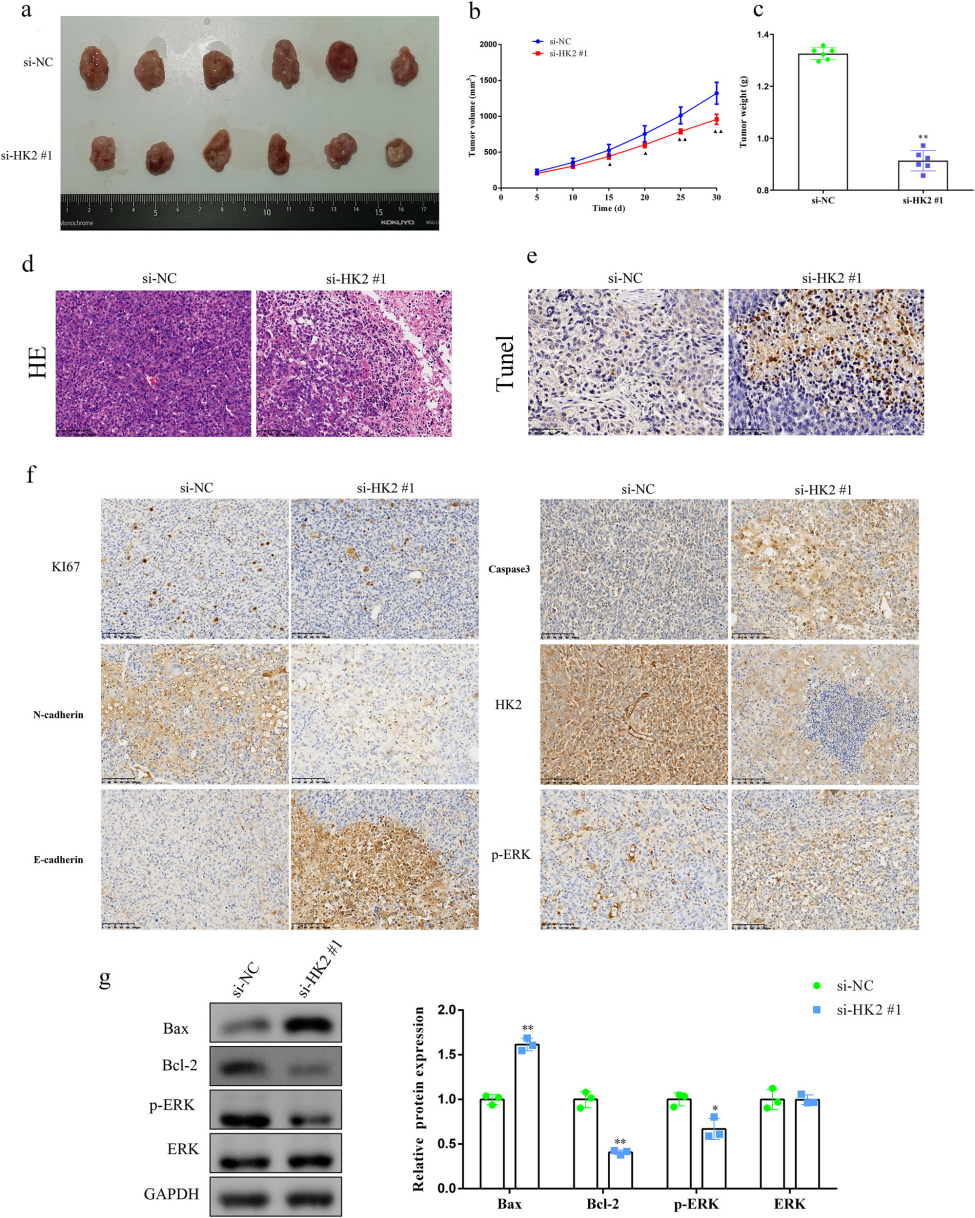
HK2 knockdown suppresses DLBCL tumor growth *in vivo*. (a) Representation of tumor tissues of the xenograft in nude mice. (b) The tumor growth rate and (c) weight were markedly decreased in HK2-knockdown cell-treated nude mice. (d) Pathologic changes and (e) apoptosis of tumor tissues were measured with hematoxylin and eosin staining (scale bar = 100 μm) and TUNEL assay (scale bar = 50 μm), respectively. (f) Ki67, caspase 3, N-cadherin, HK-2, E-cadherin, and p-ERK expression in the xenografts was measured using IHC staining (scale bar = 100 μm). (g) Expression levels of Bax, Bcl-2, p-ERK, and ERK were measured using Western blotting. *P < 0.05 and **P < 0.01 versus the si-NC group.

To confirm the role of HK2 in tumor growth *in vivo*, U2932 cells with HK2 knockdown and the corresponding negative controls were subcutaneously injected into the flanks of BALB/c nude mice. In the tumor model, HK2 silencing led to a lower growth rate of the xenograft compared with normal HK2 expression (Figure 5a and b). Coincidently, xenograft tissues from mice injected with HK2-silenced cells showed a lower weight than those from mice injected with si-NC cells (Figure 5c). Additionally, HK2 knockdown promoted xenograft tissue necrosis and apoptosis compared, as shown by HE and TUNEL staining, respectively (Figure 5d and e). Furthermore, the IHC analysis of xenograft tissues revealed that Ki67, N-cadherin, HK2, and p-ERK expression was decreased, while caspase-3 and E-cadherin expression was increased in the HK2-knockdown group (Figure 5f). Moreover, the results of Western blotting also demonstrated that knockdown of HK2 could markedly suppress the expression of Bcl-2 and p-ERK and increase the expression of Bax in tumor tissues (Figure 5g). To conclude, these data confirmed that HK2 could promote tumor growth *in vivo* and may have an effect on the ERK signaling pathway.

## 4 Discussion

DLBCL is the most common lymphoid malignancy in approximately 30% of all NHL cases worldwide [29]. Although remarkable progress had been achieved in the recent several years and nearly 60–70% of all DLBCL patients experience satisfactory clinical outcomes, the potential mechanism of DLBCL remains obscure [4]. Hence, promising targets must be investigated to deepen our understanding of the physiological and pathological processes of DLBCL. Recent studies have illustrated that HK2 is closely associated with the pathology of B-cell malignant lymphoma. For instance, Nakajima et al. [30] reported that HK2 levels were significantly elevated in B-cell lymphoma cells through c-MYC and HIF; HK2 was further overexpressed under a hypoxic microenvironment, triggering cisplatin resistance in B-cell lymphoma [30]. More importantly, HK2 inhibition by the HDAC inhibitor panobinostat remarkably ameliorated the cisplatin resistance of B-cell lymphoma cell lines [30]. Similarly, Bhalla et al. [13] reported that HK2 may serve as a hub metabolic driver to promote the DLBCL phenotype under hypoxic stress. From these findings, HK2 can be implicated in the carcinogenesis of DLBCL.

In the present study, HK2 was significantly upregulated in DLBCL cell lines, and high HK2 expression was associated with DLBCL prognosis to a certain extent, consistent with previous reports [13,30,31]. Subsequently, we knocked down HK2 expression in U2932 and SU-DHL-4 cells and assessed its effects on cell viability, proliferation, apoptosis, migration, and invasion. HK2 knockdown significantly inhibited the proliferation, migration, and invasion but promoted the apoptosis of U2932 and SU-DHL-4 cells. Furthermore, *in vivo* experiments suggested that HK2 knockdown significantly inhibited the growth of tumors derived from U2932 cells. These results were consistent with the conclusion of a previous genetic knockdown experiment that HK2 is a direct contributor to DLBCL development [13]. Interestingly, in a conditional knockout mouse model, HK2 mediated KRas-driven lung carcinoma and ErbB2-driven generation and maintenance of breast cancer, and HK2 downregulation inhibited lung cancer *in vitro* and *in vivo* as well as the malignant biological behavior of breast cancer [32]. Additionally, HK2 is valuable as a prognostic biomarker in patients with DLBCL and may be useful as a tool to assess disease risk [33]. Taken together, our findings and previous reports suggest that HK2 acts as a tumor metabolic driver in the DLBCL phenotype.

To further explore the molecular mechanism underlying HK2-mediated effects on DLBCL cell proliferation, apoptosis, migration, and invasion, we analyzed downstream effectors. ERK1 and ERK2, which are key protein kinases in Ras-Raf-MEK-ERK-MAPK signaling, are associated with apoptosis, cell proliferation, and immune response in cancer, and ERK inhibitors have been used in early clinical trials to treat various advanced/metastatic solid tumors [34]. Hicks et al. [35] found that combined BRAF (dabrafenib) and ERK1/2 (SCH772984) inhibition synergistically attenuated cell growth and promoted cell apoptosis in thyroid cancer. Furthermore, Cui et al. [36] showed that FR180204, an ERK inhibitor, effectively reversed cell growth in HK2-overexpressing SiHa-HK2 and HeLa-HK2 cells. In the present study, FR180204 significantly inhibited U2932 and SU-DHL-4 cell proliferation, migration, and invasion but promoted cell apoptosis. Consistently, increased E-cadherin, Bax, and caspase-3 protein levels and decreased N-cadherin and Bcl-2 protein levels were observed in HK2-knockdown and FR180204-treated U2932 and SU-DHL-4 cells. Additionally, decreased p-ERK1/2 protein levels were observed in U2932 and SU-DHL-4 cells and xenograft tumor tissues derived from HK2-knockdown U2932 cells. Taken together, these results suggest that HK2 overexpression activates ERK1/2 signaling in U2932 and SU-DHL-4 cells. However, the present study has a limitation. Further research is warranted to confirm the potential molecular regulatory mechanism through which HK2 upregulates ERK1/2 expression in DLBCL cell lines.

## 5 Conclusions

The present study demonstrated that HK2 knockdown inactivates ERK1/2 signaling to inhibit cell proliferation, migration, and invasion but promotes cell apoptosis *in vitro* and *in vivo* by inducing E-cadherin, Bax, and caspase-3 protein expression and suppressing Ki67, N-cadherin, and Bcl-2 protein expression in DLBCL.
